# Syntabulin regulates the trafficking of PICK1-containing vesicles in neurons

**DOI:** 10.1038/srep20924

**Published:** 2016-02-12

**Authors:** Junyu Xu, Na Wang, Jian-hong Luo, Jun Xia

**Affiliations:** 1 Department of Neurobiology, Key Laboratory of Medical Neurobiology of Ministry of Health, Zhejiang Province Key Laboratory of Neurobiology, Zhejiang University School of Medicine, Hangzhou, Zhejiang, 310058, P.R. China; 2 Division of Life Science, Division of Biomedical Engineering, and State Key Laboratory of Molecular Neuroscience, The Hong Kong University of Science and Technology, Clear Water Bay, Kowloon, Hong Kong, China

## Abstract

PICK1 (protein interacting with C-kinase 1) is a peripheral membrane protein that interacts with diverse membrane proteins. PICK1 has been shown to regulate the clustering and membrane localization of synaptic receptors such as AMPA (α-amino-3-hydroxy-5-methyl-4-isoxazolepropionic acid) receptors, metabotropic glutamate receptor 7, and ASICs (acid-sensing ion channels). Moreover, recent evidence suggests that PICK1 can mediate the trafficking of various vesicles out from the Golgi complex in several cell systems, including neurons. However, how PICK1 affects vesicle-trafficking dynamics remains unexplored. Here, we show that PICK1 mediates vesicle trafficking by interacting with syntabulin, a kinesin-binding protein that mediates the trafficking of both synaptic vesicles and mitochondria in axons. Syntabulin recruits PICK1 onto microtubule structures and mediates the trafficking of PICK1-containing vesicles along microtubules. In neurons, syntabulin alters PICK1 expression by recruiting PICK1 into axons and regulates the trafficking dynamics of PICK1-containing vesicles. Furthermore, we show that syntabulin forms a complex with PICK1 and ASICs, regulates ASIC protein expression in neurons, and participates in ASIC-induced acidotoxicity.

PICK1 (protein interacting with C-kinase 1) is a peripheral membrane protein expressed mainly in the brain, testis, and pancreas^1^,^2^,^3^. PICK1 has been found to interact and cocluster with numerous membrane receptors^4^, including AMPA (α-amino-3-hydroxy-5-methyl-4-isoxazolepropionic acid) receptors^1^,^2^, mGluR7 receptors^5^,^6^,^7^, and acid-sensing ion channels (ASICs)^8^,^9^,^10^,^11^. Furthermore, PICK1 plays key roles in regulating the surface expression and proper functioning of these critical receptors in synaptic transmission, LTP (long-term potentiation), LTD (long-term depression), and low pH-mediated cell toxicity^12^,^13^,^14^,^15^,^16^.

Cumulative evidence suggests a crucial role of PICK1 in vesicle trafficking. PICK1 is expressed at high level in the brain, testis, kidney, stomach, and pancreas, where high levels of vesicle secretion occur^1^,^2^,^3^. Moreover, PICK1 knockout (KO) mice show severe symptoms related to defects in vesicle formation and trafficking: PICK1 KO mice exhibit both impaired LTD and LTP, during which AMPA receptors are endocytosed from or incorporated into synapses^14^,^17^. The KO mice are also infertile as a result of globozoospermia^18^. PICK1 has also been shown to interact with Golgi-associated PDZ (PSD-95/Dlg/ZO-1)- and coiled-coil motif-containing protein (GOPC) and protein kinase 2 subunit CK2α′, and regulate vesicle trafficking from the Golgi apparatus to the acrosome in acrosome biogenesis^18^. Furthermore, PICK1 associates with insulin granules in pancreatic β-cells^19^. The formation of a heteromeric or homomeric PICK1 complex with its interaction partner ICA69 (islet cell autoantigen 69) determines its association with proinsulin granules or mature insulin granules. Loss of PICK1 and ICA69 leads to impaired conversion of proinsulin to mature insulin, and PICK1 KO mice display diabetes-like symptoms such as glucose intolerance, insufficient insulin release, and elevated proinsulin secretion^19^. Moreover, both PICK1-deficient *Drosophila* and mice display somatic growth retardation due to impaired biogenesis of growth hormone secretory vesicles^20^. Our previous studies in neurons also showed that PICK1 could maintain a synaptic pool of AMPA receptors that might exist in a clustered vesicle form^21^,^22^.

PICK1 protein features a unique combination of a PDZ domain and a BAR (Bin/amphiphysin/Rvs) domain. The PDZ domain of PICK1 interacts with membrane proteins such as GluA2, and BAR domains in proteins form banana-shaped dimers that sense the curved membrane on vesicles^23^. The combination of a PDZ domain and a BAR domain in PICK1 makes the protein capable of coupling membrane proteins to trafficking vesicles. For example, in the case of AMPA receptors, PICK1 binds to the GluA2 subunit in a PDZ-dependent manner^1^,^2^, and the BAR domain of PICK1 binds to lipids and targets the proteins to synapses or dendritic shafts by forming distinct dimeric complexes^3^,^22^. As a result of this synergetic effect of the above mentioned two domains, PICK1 mediates the translocation of AMPA receptors into and out of synapses^21^,^22^.

Although PICK1 serves as a linker for the binding of vesicle cargo proteins and vesicle targeting, the trafficking route through which PICK1 transports the bound receptors remains unknown, as does the underlying trafficking mechanism. To address these questions, we performed yeast-two-hybrid screening by using PICK1 as the bait and identified a microtubule trafficking-associated protein, syntabulin/golsyn (hereafter syntabulin), as a previously unrecognized PICK1-interacting partner. Further investigation revealed that syntabulin recruits PICK1 onto microtubule structures and mediates microtubule-dependent movement of PICK1-containing vesicles, and that syntabulin regulates the axonal targeting, clustering, and retrograde trafficking of PICK1-containing vesicles. Lastly, we report that syntabulin contributes to PICK1-mediated surface expression of ASIC2 and ASIC-mediated acidotoxicity.

## Results

### Syntabulin interacts with PICK1

**Figure 1:**
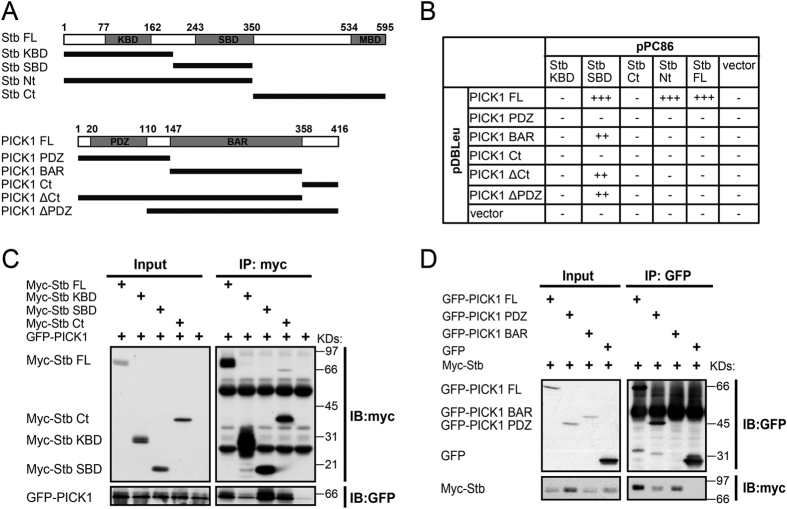
Characterization of syntabulin-PICK1 interaction. (**A**) Diagram of syntabulin and PICK1 proteins. The amino acid numbers are indicated above the bar diagram, and the coverage of partial domains is indicated below. Stb, syntabulin; FL, full-length; KBD, kinesin-binding domain; SBD, syntaxin-binding domain; MBD, mitochondrion-binding domain; Nt, N-terminus; Ct, C-terminus. (**B**) Yeast-two-hybrid result of syntabulin-PICK1 interaction domain mapping: −, no clear yeast colony growing on SCM-3 plates; ++, positive colonies growing on SCM-3 plates; +++, positive colonies growing on SCM-4 plates. The result shows an interaction between syntabulin SBD and PICK1 BAR. (**C**) Coimmunoprecipitation of syntabulin domain fragments and PICK1 from lysates of HEK293T cells overexpressing these proteins. The results show a strong interaction between syntabulin SBD and PICK1. (**D**) Coimmunoprecipitation of PICK1 domain fragments and syntabulin overexpressed in HEK293T cells. The results show that PICK1 BAR domain exhibits a strong binding.

To identify potential PICK1-binding partners, we performed yeast-two-hybrid screening on a rat brain cDNA library by using PICK1 as the bait. A part of the protein coded by the syntabulin gene was screened out as one of several PICK1-binding proteins. Syntabulin belongs to a family of proteins that are involved in kinesin and mitochondrion binding and vesicle trafficking^24^,^25^,^26^, dorsal axis formation and stimulated insulin secretion^27^,^28^,^29^. Syntabulin was reported to contain three main functional domains: the N-terminal kinesin-binding domain (KBD), the central syntaxin-binding domain (SBD), and the C-terminal mitochondrion-binding domain (MBD)^24^,^25^,^26^. We generated truncated forms of PICK1 and syntabulin that contained distinct functional domains (Fig. 1A). Using these constructs, we mapped the interacting domains between PICK1 and syntabulin by using the yeast-two-hybrid system. Our results showed that the BAR domain of PICK1 mainly mediates the syntabulin binding and that the syntabulin central SBD is responsible for PICK1 binding (Fig. 1B).

Next, we confirmed the interaction between PICK1 and syntabulin in a mammalian system by performing coimmunoprecipitation assays: GFP-tagged PICK1 was transfected into HEK293T cells together with full-length or truncated myc-tagged syntabulin, and the result showed that syntabulin SBD was mainly responsible for PICK1 binding (Fig. 1C). We also performed reverse coimmunoprecipitation assays in which myc-tagged syntabulin was transfected into HEK293T cells together with full-length or truncated GFP-tagged PICK1; the results of these assays showed that both the PDZ domain and the BAR domain of PICK1 interacted with syntabulin, with the BAR domain exhibiting a higher interaction affinity (Fig. 1D). Unfortunately, we were unable to detect the endogenous interaction in mouse brain homogenate or cultured hippocampal neurons using either syntabulin or PICK1 antibodies. As syntabulin and PICK1 are involved in vesicle trafficking, we think the association and dissociation between the two proteins might be too dynamic to be detected by the CoIP assay. Neuronal activities might also be involved in regulating this interaction. Nevertheless, our results from HEK293T cells suggest that the syntabulin-PICK1 interaction is mediated by the SBD of syntabulin and mainly the BAR domain of PICK1.

### Syntabulin regulates PICK1 microtubule localization and transport

**Figure 2:**
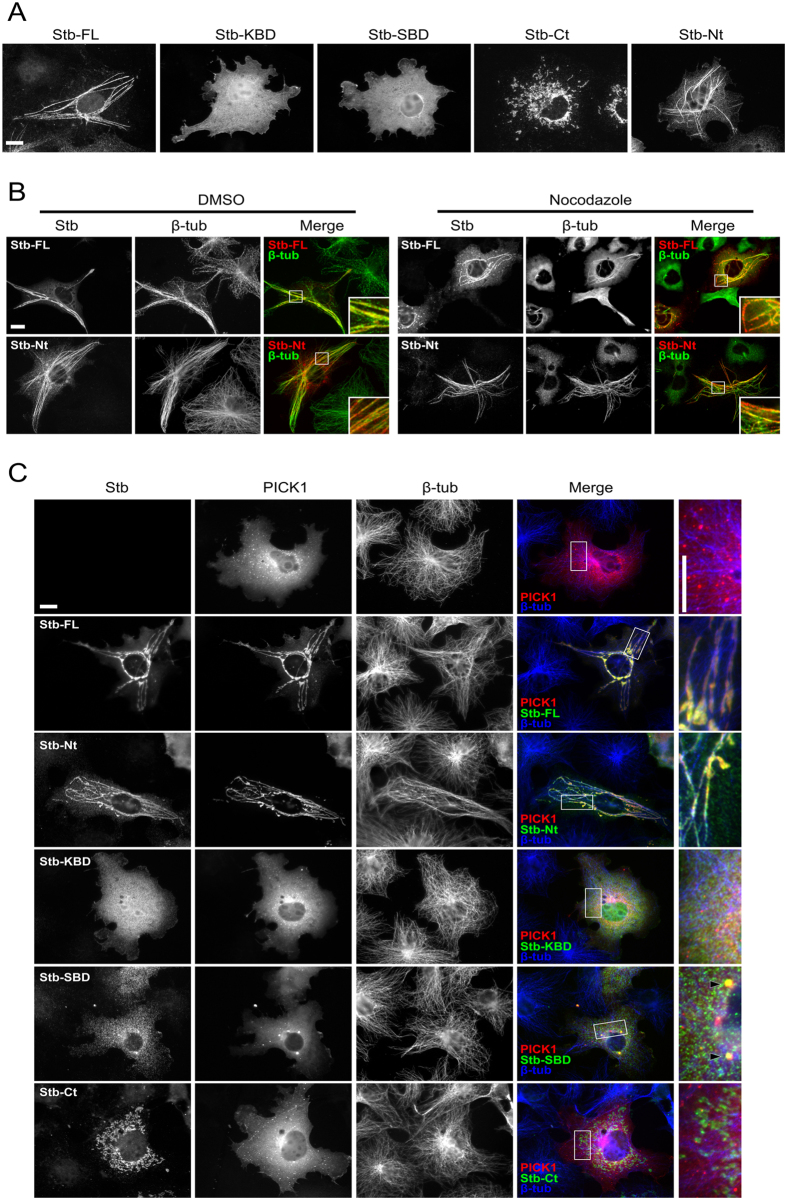
Syntabulin recruits PICK1 onto microtubule structures. (**A**) Expression pattern of myc-tagged syntabulin full-length or partial fragments in COS7 cells. (**B**) Myc-Syntabulin-FL or the N-terminal fragment containing both KBD and SBD can induce microtubule bundles. β-tub, β-tubulin. (**C**) COS7 cells were transfected with GFP-PICK1 together with or without myc-Syntabulin-FL/partial-fragment constructs, and then stained with myc and β-tubulin antibodies. The results show that Syntabulin-FL and -Nt fragments were able to recruit PICK1 onto microtubule structures. The SBD of syntabulin formed coclusters (arrowheads) with PICK1 and showed no clear relationship with microtubules. Scale bar = 10 μm in all panels.

To further explore syntabulin’s functional effect on PICK1, we first examined the localization of syntabulin by overexpressing myc-tagged full-length syntabulin or syntabulin domain fragments in COS7 cells. Myc-tag staining revealed that whereas Syntabulin-FL and Syntabulin-Nt showed a microtubule bundle-like distribution, Syntabulin-KBD and Syntabulin-SBD showed diffuse cytosolic distribution (Fig. 2A) and Syntabulin-Ct displayed a mitochondrial distribution (Fig. 2A and Suppl. Fig. 1).

Because microtubule bundles are nocodazole-resistant, we confirmed the distribution of Syntabulin-FL and Syntabulin-Nt by performing nocodazole treatment and β-tubulin immunostaining. Under control conditions, both Syntabulin-FL and Syntabulin-Nt colocalized with the bundle-like tubulin structure (Fig. 2B, DMSO). After 30-min nocodazole treatment, the thin microtubule structures were all disrupted, but the microtubule bundles that were revealed by β-tubulin staining remained associated with Syntabulin-FL and Syntabulin-Nt (Fig. 2B, Nocodazole). Therefore, Syntabulin-FL and Syntabulin-Nt localized on microtubule bundle structures. Moreover, Syntabulin-FL and Syntabulin-Nt were able to induce microtubule bundle formation: examination of untransfected cells showed that they contained only thin microtubule filaments and no bundle structures (Fig. 2B).

Next, we examined syntabulin’s role in regulating PICK1 distribution by coexpressing syntabulin and PICK1 in COS7 cells. When PICK1 was expressed alone in COS7 cells, it formed small cytosolic puncta that displayed no clear microtubule localization pattern (Fig. 2C). Notably, coexpressed Syntabulin-FL and Syntabulin-Nt recruited PICK1 to microtubule bundle structures (Fig. 2C, Stb-FL and Stb-Nt), whereas Syntabulin-KBD and Syntabulin-Ct, which do not interact with PICK1, exerted no effect on PICK1 distribution (Fig. 2C, Stb-KBD and Stb-Ct). Lastly, Syntabulin-SBD, which can bind PICK1, formed a few large coclusters with PICK1 (Fig. 2C, Stb-SBD). These results revealed that syntabulin regulates PICK1’s microtubule distribution through the synergetic effect of its KBD and SBD.

**Figure 3:**
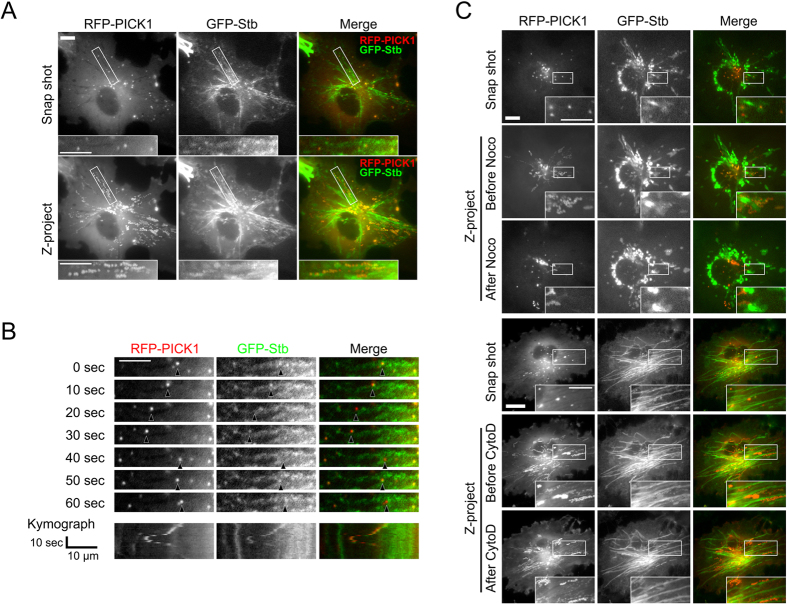
Syntabulin and PICK1 comigrate along microtubule structures. (**A**) COS7 cells were transfected with GFP-Syntabulin and RFP-PICK1 and examined under a live-imaging microscope with both green- and red-fluorescent channels; images were collected for 2 min at 2-s intervals. The “snapshot” shows the colocalization of PICK1 and syntabulin at a single time point, and the Z-projection shows the movement trajectories of PICK1 and syntabulin over 2 min. The merged image shows that the trajectory of PICK1 overlapped with that of syntabulin, which was juxtaposed to syntabulin-induced microtubule bundles. (**B**) PICK1 and syntabulin vesicle movement at different time point in the cell presented in (**A**). Arrowheads indicate the position of a single PICK1-syntabulin-containing vesicle at 10-s intervals. The kymograph also shows the overlapped movement trace of PICK1 and syntabulin. (**C**) PICK1-syntabulin-containing vesicles move in a microtubule-dependent but not actin-dependent manner. The snapshots show the original position of PICK1 and syntabulin proteins before drug treatment, and the Z-projection shows the movement trajectories of both proteins before and after drug treatment. Noco, nocodazole; CytoD, cytochalasin D. Scale bar = 10 μm in all panels.

Syntabulin was previously reported to regulate the microtubule-dependent transport of syntaxin-1 cargo vesicles, mitochondria, and active-zone components along neurites^24^,^25^,^26^; therefore, we tested whether syntabulin was also responsible for the trafficking of PICK1-containing vesicles. First, we examined syntabulin’s regulatory role in PICK1 trafficking in COS7 cells because these cells are large and flat and contain clearly detectable microtubule structures. We cotransfected GFP-tagged Syntabulin-FL and RFP-tagged PICK1 into COS7 cells and performed time-lapse imaging on both proteins by using both red and green fluorescence channels. Unlike what was observed in the immunofluorescent staining in fixed cells, PICK1 was found to colocalize with syntabulin on small puncta but not microtubule bundles, although syntabulin was detected on both the clusters and the microtubule bundles (Fig. 3A, snapshot). One possibility was that these highly dynamic vesicle structures associated with microtubules were lost during the fixation procedure. Nevertheless, time-lapse imaging revealed that PICK1 comigrated with syntabulin and that it migrated bidirectionally (Fig. 3B and Suppl. Movie 1). When we traced the movement of PICK1 and syntabulin by using stack Z-projection, which is a simple summation of images captured at every time point, we found that the movement trajectory of PICK1-syntabulin coclusters was juxtaposed to syntabulin-containing microtubule structures (Fig. 3A, Z-project).

To confirm that the PICK1-containing vesicles moved in a microtubule-dependent manner, we treated COS7 cells with nocodazole during the time-lapse imaging. The Z-projection results showed that after 20 min of nocodazole treatment, the PICK1-syntabulin vesicles stopped moving and vibrated within a small region (Fig. 3C and Suppl. Movies 2 and 3). By contrast, when COS7 cells were exposed to cytochalasin D, an actin-bundle destabilizer, the PICK1-syntabulin vesicles continued to move dynamically even after a 40-min treatment (Fig. 3C and Suppl. Movies 4 and 5). However, when PICK1 was coexpressed with only Syntabulin-SBD, the PICK1-syntabulin coclusters did not move and exhibited only local vibration (Suppl. Movie 6). Therefore, syntabulin regulates the microtubule-dependent movement of PICK1.

### Syntabulin regulates the axonal targeting, clustering, and trafficking of PICK1

**Figure 4:**
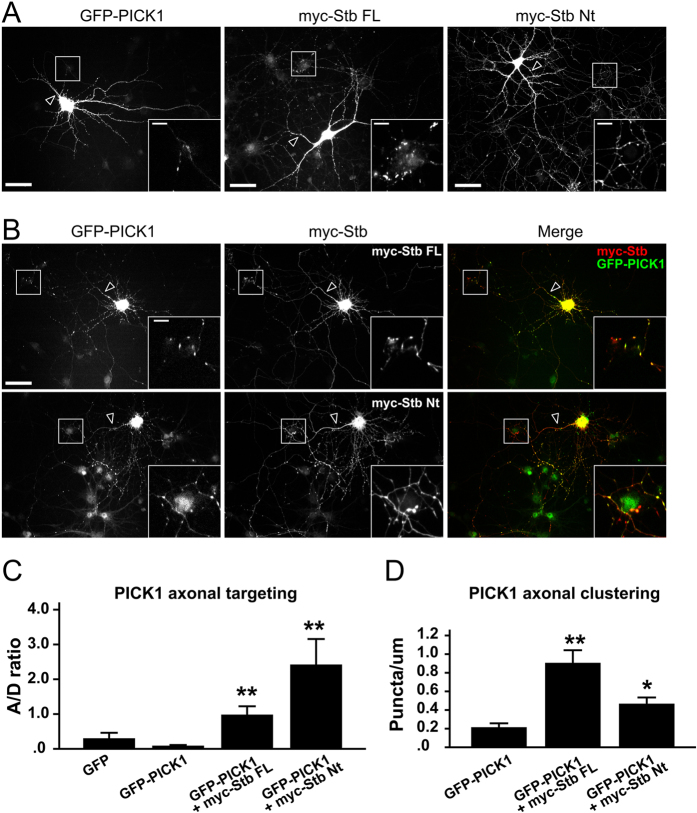
Syntabulin facilitates the axonal distribution of PICK1. (**A**) Distribution of GFP-PICK1 and myc-Syntabulin full-length and N-terminal proteins in neurons when transfected alone. The enlarged pictures show clusters in axons in contact with another neuron. Arrowheads indicate the axons. (**B**) Neurons were doubly transfected with PICK1 and syntabulin and stained with the myc antibody. The merged images show almost complete colocalization of PICK1 clusters with syntabulin in axons. The enlarged pictures show clusters in axons in contact with another neuron. Scale bar = 50 μm for the original image, 10 μm for the insets in both (**A**,**B**). Arrowheads indicate the axons. (**C**) Quantification of PICK1 axonal targeting. A/D ratio, axon/dendrite ratio. The result shows that when coexpressed with syntabulin, PICK1 axonal targeting was increased significantly. **P = 0.003 and *P* = 0.007 for GFP-PICK1 expressed together with Syntabulin-FL and Syntabulin-Nt, as compared with GFP-PICK1 expressed alone; one-way ANOVA and Sidak test, n = 3–8 independent experiments. (**D**) Quantification of PICK1 axonal clustering. Coexpression with syntabulin significantly increased PICK1 axonal clustering. **P = 0.003 and *P = 0.018 compared to GFP-PICK1; one-way ANOVA and Sidak test, n = 4 independent experiments. Error bars represent the SEM.

In order to investigate the functional effect of syntabulin on PICK1 expression and trafficking in neurons, we transfected primary hippocampal neurons with GFP-tagged PICK1 or myc-tagged syntabulin and examined their expression patterns. Syntabulin was detected both on the soma and the dendrites, but numerous, clear puncta were also observed along axons (Fig. 4A, myc-Syntabulin-FL). Moreover, Syntabulin-Nt, which contains the KBD and the SBD, exhibited strong axonal-targeting capacity, but formed fewer distinct puncta than Syntabulin-FL did (Fig. 4A, myc-Syntabulin-Nt). In hippocampal neurons, GFP-PICK1 was expressed mainly in the soma and dendrites, with enrichment in spine structures, which is consistent with previous reports^2^,^22^, and a small fraction of GFP-PICK1 was also detected in axons in a minor population of transfected neurons (Fig. 4A, GFP-PICK1). Interestingly, when coexpressed with Syntabulin-FL or Syntabulin-Nt, PICK1 was readily detected in axons and was almost completely colocalized with Syntabulin-FL and Syntabulin-Nt (Fig. 4B and Suppl. Fig. 2). We quantified the axonal enrichment of PICK1 by measuring the expression of PICK1 in axons relative to that in dendrites. The results showed Syntabulin-FL and Syntabulin-Nt increased PICK1 axonal targeting substantially and in a statistically significant manner (Fig. 4C). Furthermore, PICK1 formed considerably more puncta along axons when coexpressed with syntabulin than when expressed by itself (Fig. 4B). Quantification revealed that PICK1 clustering was significantly increased when it was coexpressed with Syntabulin-FL or Syntabulin-Nt (Fig. 4D). Comparison of PICK1 expression patterns in distinct experimental groups showed that PICK1 exhibited weaker axonal targeting but stronger axonal clustering when coexpressed with Syntabulin-FL than with Syntabulin-Nt (Fig. 4C,D). This difference could arise as a result of the nature of syntabulin protein expression: Syntabulin-FL displayed a more clustered pattern than Syntabulin-Nt did, whereas Syntabulin-Nt displayed a comparatively more diffuse distribution.

**Figure 5:**
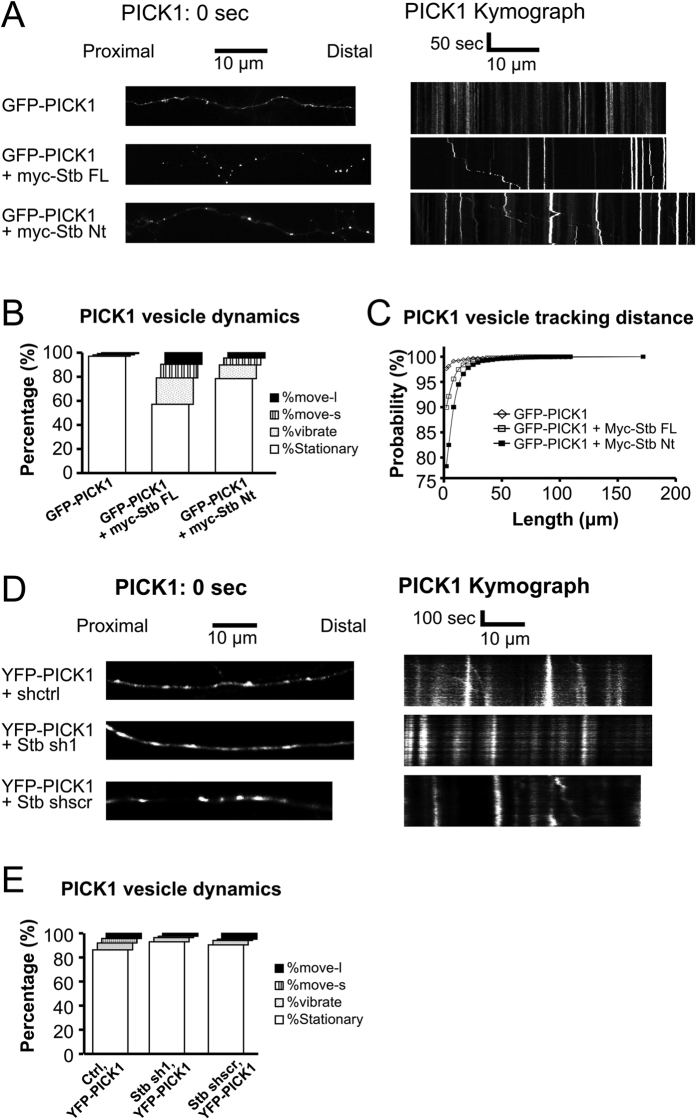
Syntabulin mediates dynamic movement of PICK1 along axons. (**A**) Time-lapse imaging of GFP-PICK1 expressed alone or together with syntabulin in neurons. Left panels show the original PICK1 distribution in axons. Right panels show the kymograph of PICK1. (**B**) Classification of PICK1 vesicles in axons. The result shows that the population of mobile PICK1 vesicles increased upon syntabulin coexpression; n = 4 independent experiments. (**C**) Cumulative probability of PICK1 distance moved in 5 min of time-lapse imaging; >1000 vesicles from 4 independent experiments were quantified. (**D**) Neurons were infected with lentivirons knocking down endogenous syntabulin and expressing YFP-PICK1 simutaneously. Time lapse imaging of YFP-PICK1 were carried out to examine the PICK1-containing vesicle dynamics. Scale bars are as indicated. (**E**) Quantification of the population of PICK1-containing vesicles. Result showed significant reduction of mobile PICK1 vesicles after syntabulin knockdown. >700 vesicles from 3 independent experiments were quantified for each group.

Next, we performed time-lapse imaging of the PICK1-containing vesicles detected along axons. GFP-tagged PICK1 localized in dendritic spines was mostly stationary (Suppl. Movie 7). PICK1-containing vesicles were detected in axons in only a few neurons, but these PICK1 vesicles were also stationary (Fig. 5A, GFP-PICK1; Suppl. Movie 8). However, when PICK1 was coexpressed with Syntabulin-FL or Syntabulin-Nt, the mobility of PICK1-containing vesicles was increased markedly and the vesicles were transported toward the distal part of axons (Fig. 5A and Suppl. Movies 9 and 10). This agrees with previous studies reporting that syntabulin interacts with KIF5B and regulates the anterograde movement of transported cargo^24^,^25^. We classified the distinct movement behaviors of PICK1-vesicles into 4 groups: stationary (no movement), vibrating (forward and backward movement within 5 μm from the original position), small-movement (5–10 μm movement from the original position), and large-movement (>10 μm movement from the original position). Quantification of the results revealed that when PICK1 was coexpressed with Syntabulin-FL and Syntabulin-Nt, the mobile PICK1 population increased substantially (Fig. 5B), and the cumulative probability plot of the data further revealed that the PICK1 movement distance was increased when it was coexpressed with syntabulin (Fig. 5C). Collectively, these results showed that syntabulin increased PICK1 axonal targeting and clustering and facilitated PICK1 trafficking along axons, and that the co-functioning of syntabulin KBD and SBD was sufficient for syntabulin to perform this regulatory function.

### Endogenous syntabulin regulates PICK1 axonal targeting and trafficking

**Figure 6:**
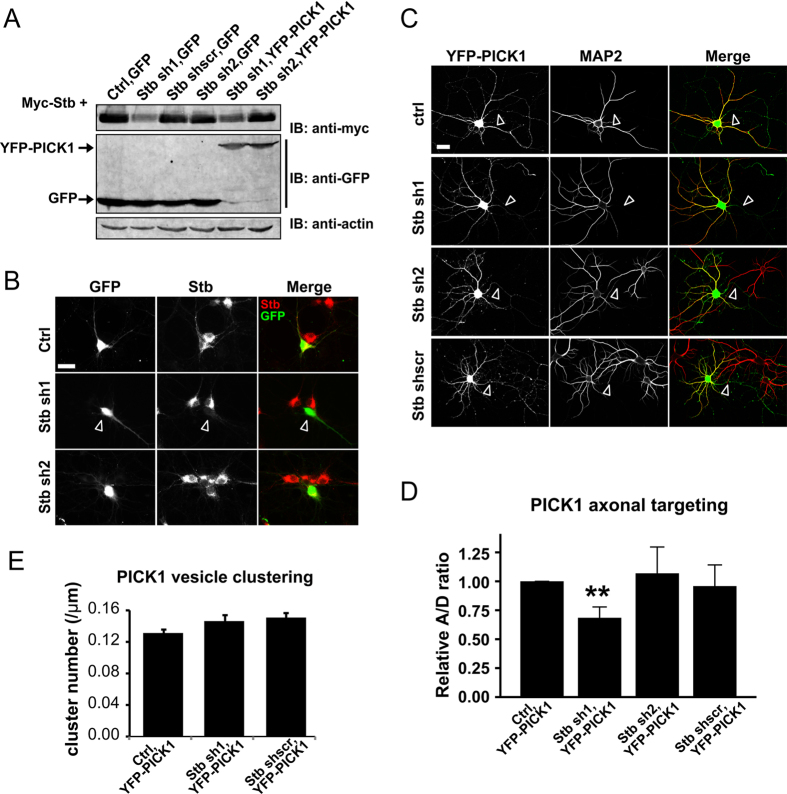
Knockdown of syntabulin in neurons affects PICK1 axonal targeting. (**A**) Test of syntabulin shRNA knockdown efficiency in HEK293T cells, which were transfected with myc-Syntabulin and the lentiviral constructs of syntabulin shRNAs. (**B**) Staining of endogenous syntabulin in neurons after shRNA viral infection for 5 days. The result shows that syntabulin expression was knocked down highly efficiently by shRNA #1. Arrowheads indicate a neuron in which syntabulin was successfully knocked down. Scale bar = 20 μm. (**C**) YFP-PICK1 distribution in neurons after syntabulin knockdown. Arrowheads indicate the axons which are MAP2 signal-free. Scale bar = 20 μm. (**D**) Quantification of PICK1 axonal targeting after syntabulin knockdown. The A/D ratios were normalized against control. The result shows a slight but significant impairment in the axonal targeting of PICK1 after syntabulin knockdown compared to control. **P = 0.008, nonparametric Kruskal-Wallis Test, n = 6 independent experiments; error bars represent the SEM. (**E**) Quantification result of axonal clustering of PICK1 after syntabulin knockdown. Result showed no significant change on PICK1 clustering after syntabulin knockdown. one-way ANOVA and Sidak test, n = 3 independent experiments. Error bars represent the SEM.

To confirm the regulatory role of endogenous syntabulin in neurons, we designed shRNAs targeting syntabulin. Western blotting analysis in a heterologous cell system showed that syntabulin shRNA #1 efficiently knocked down the protein’s expression, but the scrambled control shRNA and shRNA #2 did not (Fig. 6A). To confirm the knockdown effect in neurons, we generated a lentiviral transduction vector for the shRNAs and infected hippocampal neurons with the virus. The knockdown of endogenous syntabulin was examined using a custom-generated syntabulin antibody against the N-terminus (Suppl. Fig. 3). The result showed that shRNA #1 clearly and efficiently knocked down the expression of endogenous syntabulin (Fig. 6B). In order to concurrently observe the axonal targeting of PICK1, we added a PICK1 DNA coding sequence into the shRNA lentiviral backbone. Western blotting analysis confirmed that after viral infection, endogenous syntabulin expression was successfully knocked down and YFP-tagged PICK1 was expressed (Fig. 6A). Quantification revealed that in these syntabulin-knockdown neurons, axonal targeting of YFP-PICK1 was decreased slightly but significantly relative to that in controls (Fig. 6C,D). This could be due to the reason that the overexpression of PICK1 markedly increased the PICK1 homodimer population and spine localization, and led to the distribution of only a very small amount of PICK1 in axons. Consequently, knocking down endogenous syntabulin expression was not sufficient for causing a large change in PICK1 localization.

We then further examined the clustering as well as the dynamic of PICK1 vesicles in axons after syntabulin knockdown, despite of the low expression level of PICK1 in axons. After syntabulin knockdown, although PICK1 showed no significant change in the clustering number in axons as expected (Fig. 6E), we did detect a significant reduction in the PICK1 vesicle mobility along axons (Fig. 5D,E). This result further supports our finding that syntabulin regulates the axonal trafficking of PICK1-containing vesicles in neurons.

### Syntabulin differentially binds to ASICs through PICK1 and regulates ASIC-mediated acidotoxicity in neurons

Because syntabulin regulated the axonal targeting and trafficking of PICK1-containing vesicles, we tested whether syntabulin also functions in the axonal trafficking of PICK1-interacting receptors. ASICs are members of the pH receptor family expressed in the mammalian brain^30^,^31^,^32^ that modulate H^+^-activated currents in hippocampal neurons^33^ and are involved in triggering neuronal cell death in acidosis^34^,^35^. PICK1 interacts with ASIC2a and regulates ASIC2a currents through its PDZ domain^8^,^9^,^10^. Furthermore, PICK1 also interacts with ASIC1a^11^ and increases ASIC-mediated acidotoxicity *in vitro*^13^.

**Figure 7:**
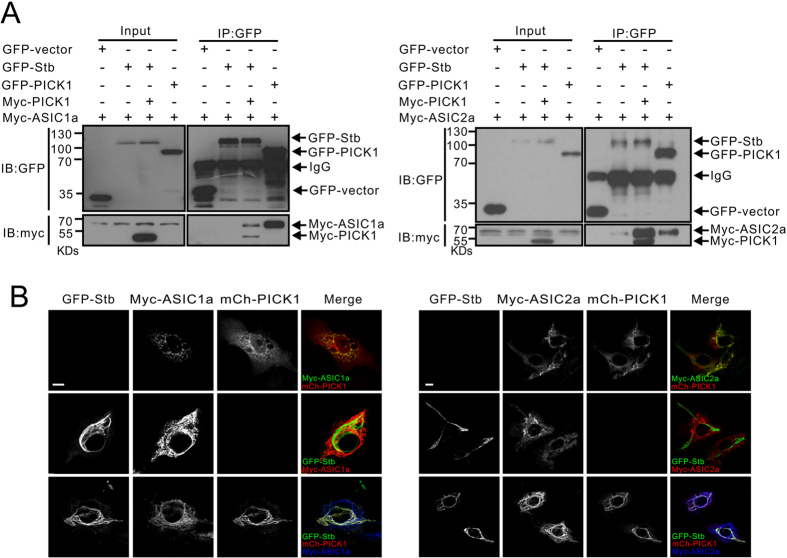
Syntabulin and PICK1 selectively form complex with ASIC2a *in vitro*. (**A**) GFP-Syntabulin, myc-ASIC2a, or myc-ASIC1a was transfected into HEK293T cells together with or without myc-PICK1, and immunoprecipitated with GFP antibody. Syntabulin showed higher binding affinity for the ASIC2a-PICK1 complex than for the ASIC1a-PICK1 complex. (**B**) COS7 cells were transfected with PICK1, ASIC1a/2a, and syntabulin in different combinations (as indicated). mch: mcherry. PICK1 and ASIC1a/2a formed numerous coclusters in the cytosol when coexpressed in COS7 cells (top panels). Syntabulin showed no clear colocalization with ASIC1a/2a (middle panels). Syntabulin, PICK1 and ASIC2a but not ASIC1a showed a microtubule distribution pattern when expressed together (bottom panels). Scale bar = 10 μm.

To investigate syntabulin’s role in ASIC trafficking and function, we first examined whether syntabulin, ASIC1/2, and PICK1 coexist in a complex by performing coimmunoprecipitation assays. The results showed that PICK1, but not syntabulin, interacted with ASIC1a/2a; when PICK1 was present, syntabulin was efficiently immunoprecipitated with ASIC2a, but it bound only weakly to ASIC1a (Fig. 7A); this indicated the selective formation of a syntabulin-PICK1-ASIC2a complex. Next, we examined the expression pattern of ASICs in COS7 cells in presence and absence of PICK1 and syntabulin. Both ASIC1a and ASIC2a formed coclusters with PICK1 but showed a distinct localization relative to syntabulin (Fig. 7B). However, when ASICs and syntabulin were expressed in COS7 cells together with PICK1, ASIC2a but not ASIC1a was translocated onto microtubule bundle structures (Fig. 7B). Therefore, PICK1 functions as a selective linker between syntabulin and ASICs, and recruits ASIC2a onto microtubule structures.

**Figure 8:**
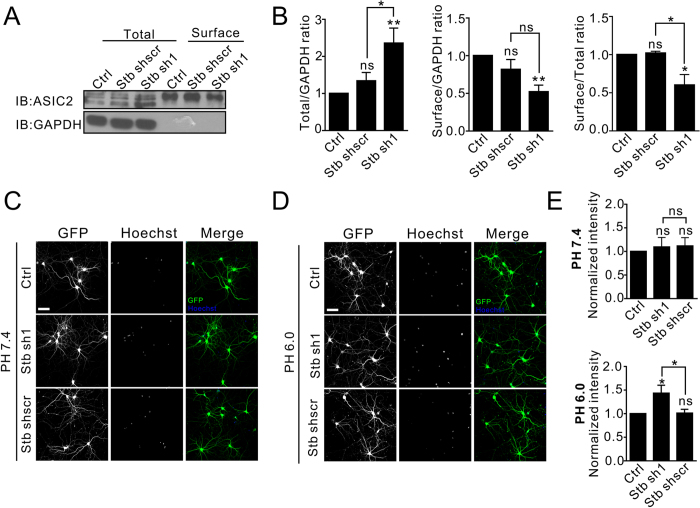
Syntabulin affects ASIC2 expression levels and regulates neuronal toxicity. (**A**) Neurons were infected with syntabulin shRNA lentivirus as indicated at DIV5 and examined at DIV13. Syntabulin knockdown significantly increased total ASIC2 expression but reduced surface ASIC2 levels as compared with the levels in control and scrambled-shRNA control. (**B**) Quantification of normalized ASIC2/GAPDH ratio, total ASIC2/surface ASIC2 ratio and surface ASIC2 levels. One-way ANOVA and Sidak test; **P = 0.008, and *P* = 0.009 for Stb sh#1 Total ASIC2/GAPDH and Surface ASIC2/GAPDH compared to control respectively; *P = 0.044 and *P = 0.016 for Stb #1 total ASIC2/GAPDH and Surface/Total ASIC2 ratio compared to scrambled shRNA control respectively; *P = 0.020 for Stb #1 Surface/Total ASIC2 ratio compared to control; “ns” *P* > 0.05. n = 4 − 6 independent experiments. Error bars represent the SEM. (**C**,**D**) Hoechst staining of neurons after syntabulin knockdown, in pH 7.4 solution and pH6.0 solution. Knockdown of syntabulin significantly increased cell death as compared with controls in pH6.0. Scale bar = 20 μm. (**E**) Quantification of normalized total Hoechst intensity. One-way ANOVA and Sidak test; *P = 0.035 compared to control; *P = 0.043 compared to scrambled-shRNA control; “ns” *P* > 0.05; n = 6 independent experimental repeats. Error bars represent the SEM.

To examine syntabulin’s regulatory effect on ASIC2 in neurons, we used lentiviral infection to knockdown endogenous syntabulin expression and then checked ASIC2 levels. Both western blots and quantification of the blotting data showed that the total ASIC2 level in neurons was significantly increased after syntabulin knockdown (Fig. 8A,B). However, surface-biotinylation assays revealed that the expression of membrane ASIC2 was decreased significantly (Fig. 8A,B). Despite of the lack of significance in scrambled syntabulin shRNA compared to shRNA #1, which may be caused by the high variation of the scramble shRNA effect, we detected significant reduction in surface/total ratio of ASIC2 (Fig. 8A,B). Therefore, a reduction in endogenous syntabulin expression could cause a lowering of the surface expression of ASIC2, and the increase in the total ASIC2 level could represent a compensation for the reduction in surface ASIC2 expression.

ASICs are critical for sensing extracellular acidic environments and these channels mediate acidosis-induced cell death. Thus, we investigated whether syntabulin plays a role in acidotoxicity. First, we knocked down endogenous syntabulin by using lentiviral infection, and then challenged the neurons with distinct pH environments. Hoechst staining was used to identify neurons undergoing apoptosis^13^,^36^,^37^. When neurons were incubated in a pH 7.4 medium, cell viability was similar among the control and syntabulin-knockdown groups (Fig. 8C,E). However, at pH 6.0, apoptotic events were significantly increased in the syntabulin-knockdown group as compared with that in the control group and the scrambled-shRNA control group (Fig. 8D,E). This result suggests that syntabulin participates in ASIC-mediated acidotoxicity in neurons.

## Discussion

PICK1 is a unique protein that possesses both a PDZ domain and a BAR domain. Through the PDZ domain, PICK1 interacts with several membrane receptor proteins such as AMPA receptor, mGluR7 receptor, dopamine transporter, and ASIC, and through the BAR domain, PICK1 can bind to lipids and form cargo vesicles containing the receptors. The synergetic effect of the PDZ and BAR domains of PICK1 ensures PICK1-dependent regulation of vesicle-mediated receptor trafficking from and to synapses. Conversely, the binding of distinct interacting partners further expands PICK1’s regulatory capability. Previously, we showed that whereas the PICK1-ICA69 heteromeric complex regulates AMPA receptor trafficking from the soma to the dendritic shaft in neurons, the PICK1-PICK1 homomeric complex regulates AMPA receptor trafficking into synapses^3^. Furthermore, the PICK1-F-actin-Arp2/3 complex regulates NMDA-induced AMPA receptor internalization^38^. These results indicate that by forming different trafficking complexes, PICK1 might be able to traffic distinct receptors through divergent mechanisms; however, the medium through which PICK1-containing vesicles are transported remains unknown. Moreover, an electron microscopy study revealed that PICK1 localizes at presynaptic terminals^39^, and thus the presynaptic clustering of mGluR7 and GluA2 could be a functional consequence of this specific PICK1 targeting^5^,^39^, although the mechanism underlying this axonal targeting is also unclear.

In this study, we used a yeast-two-hybrid screening system and identified a previously unrecognized PICK1-interacting protein, syntabulin. Syntabulin, which is involved in primary open-angle glaucoma^40^,^41^, is also named golsyn (human Golgi-localized syntaphilin-related protein)^42^. Syntabulin is a kinesin motor adaptor and it binds to syntaxin and mitochondria^24^,^25^,^26^,^43^. Through the kinesin-binding and syntaxin-binding actions of its KBD and SBD, syntabulin can regulate the movement of syntaxin-cargo vesicles along microtubule structures in neuronal processes and the anterograde transport of active-zone components along developing axons^24^,^26^. Moreover, syntabulin recruits mitochondria through its C-terminal MBD and mediates the anterograde transport of mitochondria in axons^25^. Therefore, syntabulin is highly likely to function as a key factor that regulates PICK1 movement and axonal targeting in neurons.

Based on our domain-mapping results, we identified the PICK1 BAR domain and the syntabulin SBD as the domains that mediate the interaction between these 2 proteins. We also found that in COS7 cells, syntabulin recruits PICK1 onto microtubule bundle structures and regulates the microtubule-dependent trafficking of PICK1-containing vesicles. In neurons, syntabulin facilitates the axonal targeting and clustering of PICK1, and regulates the anterograde transport of PICK1-containing vesicles. These functions of syntabulin likely result from the synergetic effect of its KBD and SBD that ensures syntabulin-PICK1 binding and PICK1 microtubule association. Full-length syntabulin contains a mitochondrion-targeting sequence and regulates the movement of mitochondria along axons^25^, and we found that when PICK1 was coexpressed with syntabulin, it colocalized with mitochondria in both COS7 cells and neurons (data not shown) and was closely associated with mitochondria during trafficking dynamics (data not shown). Thus, uncovering the relationship between PICK1 and mitochondria should be of interest: PICK1 could potentially regulate the targeting of certain mitochondrial membrane proteins, or the movement of PICK1-containing vesicles could require a “power supply” from mitochondria.

We also determined in this study that syntabulin differentially associates with the PICK1-ASIC complex. Although PICK1 was reported to bind both ASIC1a and ASIC2a^8^,^10^,^11^,^13^,^44^, syntabulin interacted with PICK1 and ASIC2a, but not with PICK1 and ASIC1a, and a knockdown of endogenous syntabulin led to a reduction in the surface ASIC2 level and an increase in the total ASIC2 level. Cell-viability measurement revealed that apoptosis under an acidic environment was significantly increased when syntabulin was knocked down in neurons. All of these results indicate that syntabulin regulates ASIC surface expression and ASIC-mediated acidotoxicity. In contrast to our results, previous reports showed that increased surface ASIC1 levels lead to acidotoxicity^13^,^45^. Our explanation is that the functional ASIC in the brain is formed of homotrimers or heterotrimers of ASIC subunits. ASIC1a and ASIC2a could form heterotrimers or homotrimers featuring distinct compositions and stoichiometry^33^,^34^,^46^,^47^. Whereas ASIC2 is Na^+^ permeable, ASIC1 is Na^+^ and Ca2^+^ permeable and contributes to calcium influx and acid-induced toxicity^48^,^49^; ASIC2 functions in neurons as a modulator for pH sensitivity^33^. Thus, a reduction in ASIC2 surface levels could cause a shift of the ASIC composition from heterotrimer to ASIC1 homotrimer and increase the calcium permeability of ASICs under a low-extracellular-pH environment, and, consequently, increase acidotoxicity. The function of ASICs is not limited to mediating acidotoxicity: ASICs also contribute to synaptic transmission, synaptic plasticity, and learning and memory^50^,^51^,^52^. Therefore, future studies should investigate syntabulin’s regulatory role in ASIC-mediated synaptic activities.

Syntabulin was also recently shown to be expressed in pancreatic β-cells and to regulate glucose-stimulated insulin secretion in a microtubule-dependent manner^28^. Intriguingly, PICK1-ICA69 and PICK1-PICK1 complexes were recently reported to mediate insulin-vesicle maturation along the insulin-secretion pathway in β-cells^19^,^20^. Thus, it should be of interest to test whether syntabulin mediates insulin secretion through PICK1.

## Methods

### cDNA constructs

Rat PICK1 full-length (FL) (residues 1–416), PICK1-PDZ (1–146), PICK1-BAR (147–358), and PICK1-Ct (358–416) in the vector pDBLeu, which contains the GAL4 DNA-binding domain, were described previously^3^. PICK1-ΔCt (residues 1–379) and PICK1-ΔPDZ (147–416) were subcloned into pDBLeu. Syntabulin-FL (residues 1–595), Syntabulin-Nt (residues 1–350), Syntabulin-KBD (1–202), Syntabulin-SBD (203–350), and Syntabulin-Ct (351–595) were amplified from human FLJ20366 DNA sequence and subcloned into the vector pPC86, which contains the GAL4 activation domain. Myc- and GFP-tagged PICK1 in myc-pRK5 and pEGFPC3 vectors were previously described^22^. RFP- and mCherry-tagged PICK1 were subcloned into pRFP-C3 or pmCherry-C3 vectors. The pRFP-C3 and pmCherry-C3 vectors were constructed by replacing the GFP sequence in the pEGFP-C3 vector with RFP and mCherry sequences, respectively. Myc- and GFP-tagged Syntabulin-FL for partial domain constructs were subcloned into myc-pRK5 and pEGFPC3 vectors. Human ASIC1a and ASIC2a constructs in pGW6B and pGW4–2b vectors were kindly provided by Dr. Garcia-Anoveros (Northwestern University) and Dr. Corey (Harvard Medical School)^9^. The myc-tag sequence was then inserted into ASIC1a and ASIC2a sequences after amino acid 120 by using a site-directed PCR method.

Syntabulin shRNA constructs were generated by annealing synthetic primer pairs and subcloned into the pSuper vector (gift from Dr. Ip, Hong Kong University of Science and Technology), and then subcloned into the pFUGW-super vector^53^ for virus generation. For concurrent PICK1 overexpression and syntabulin knockdown, the YFP-PICK1 sequence was subcloned into the viral plasmids upstream of the GFP sequence, because a stop codon was present before the GFP sequence and thus the expression of the original GFP was prevented. The HIV-1 packing vector Δ8.9 and the VSVg envelope glycoprotein plasmid were gifts from Dr. C. Lois (MIT). The annealing primers for syntabulin shRNAs were the following: #1 5′-GATCTCCaagataaaggcattcagaaTTCAAGAGAttctgaatgcctttatcttTTTTTGGAAC-3′ 5′-TCGAGTTCCAAAAAaagataaaggcattcagaaTCTCTTGAAttctgaatgcctttatcttGGA-3′ #2 5?-GATCTCCtctgaaatcatggagctcaTTCAAGAGAtgagctccatgatttcagaTTTTTGGAAC-3? 5′-TCGAGTTCCAAAAAtctgaaatcatggagctcaTCTCTTGAAtgagctccatgatttcagaGGA-3′ #1 scrambled 5′-GATCTCCtaacctgaaaggaaataagTTCAAGAGActtatttcctttcaggttaTTTTTGGAAC-3′ 5′-TCGAGTTCCAAAAAtaacctgaaaggaaataagTCTCTTGAActtatttcctttcaggttaGGA-3′

### Antibodies

The following antibodies were purchased: rabbit polyclonal ASIC2 antibody, Proteintech (17851-1-AP); mouse myc and β-tubulin antibodies, DSHB (9E10 and E7); mouse GAPDH antibody, Beyotime (GA019); mouse Map2 antibody, Sigma (M9942); and mouse β-actin antibody, Sigma (A5316). A rabbit GFP antiserum was generated by immunizing New Zealand White rabbits with a purified GST-GFP fusion protein. A rabbit syntabulin antibody was generated by injecting New Zealand White rabbits with the purified N-terminal 80 amino acids of syntabulin fused with a GST tag, and then affinity-purified by using Affigel beads (Bio-Rad, #153–6099) coupled with the purified His-tagged N80 polypeptide. We purchased horseradish peroxidase (HRP)-labeled and Cy3-conjugated secondary antibodies from Pierce, Cy3-conjugated secondary antibodies from Jackson ImmunoResearch, and Alexa 488- and 647-conjugated secondary antibodies from Invitrogen.

### Yeast two-hybrid

To map the domains that mediate the interaction between syntabulin and PICK1, the corresponding DNA constructs were cotransformed into AH109 yeast cells and grown on SCM-2 plates (lacking Leu and Trp). Positive clones were selected and tested on SCM-3 plates (lacking Leu, Trp, and His) and SCM-4 plates (lacking Leu, Trp, His and Ade).

### Coimmunoprecipitation

HEK293T cells were transfected with different plasmid combinations. Two days after transfection, cells were lysed with 0.5% Triton X-100 in Tris-buffered saline (TBS) and incubated with anti-GFP antibody/Protein A beads (GE Healthcare) at 4 °C for at least 1 h. The pelleted beads were washed once with 0.5% Triton X-100/TBS, twice with 0.5% Triton X-100/TBS plus 500 mM NaCl, and thrice with TBS, and then the captured protein complexes were eluted with 1× SDS sample buffer, boiled for 5–10 min, and analyzed by performing SDS-PAGE and immunoblotting with corresponding antibodies.

### Cell culture, transfection, and drug treatment

HEK293T and COS7 cells were cultured in MEM supplemented with 10% fetal bovine serum, 1× sodium pyruvate, and 1× penicillin-streptomycin-glutamine (all from Invitrogen). HEK293T cells were transfected using the calcium phosphate coprecipitation method, and the medium was completely changed after 9 h. COS7 cells were transfected using Lipofectamine 2000 (Invitrogen) according to manufacturer’s instruction and the medium was completely changed after 3 h. Nocodazole (Sigma, M1414) and cytochalasin D (Sigma, C8273) were prepared as 1000× stock solutions in DMSO and used at working concentrations of 5 and 2 μg/mL, respectively, for 20–40 min (as indicated).

Dissociated rat primary hippocampal neurons were cultured as described^53^. Neurons were transfected with cDNA constructs by using calcium phosphate (HEPES-buffered) coprecipitation on DIV (days *in vitro*) 5 in DMEM; after 20–30 min of calcium particle sedimentation, neurons on coverslips were washed with DMEM and transferred back into the original culture medium. All rat use procedures were approved by the Committees at Zhejiang University and Leeds University for the Care and Use of Laboratory Animals.

### Lentivirus generation

Lentiviral pFUGW constructs were transfected together with the HIV-1 packing vector Δ8.9 and VSVg envelope glycoprotein plasmid into HEK293T cells in a 2:1:1 ratio by using Lipofectamine 2000. The transfection medium was replaced with fresh medium at 3 h after transfection, and this medium was collected at 48 h after transfection to harvest the released viral particles. The medium was centrifuged at 1000 rpm at 4 °C for 5 min to remove cell debris and filtered through a 0.45-μm filter, and then aliquoted and stored at −80 °C. The viral titer was determined in HEK293T cells and neurons. Neurons were infected with the determined amounts of virus on DIV5, and treated on DIV10.

### Immunocytochemistry

COS7 cells at 24–36 h after transfection or neurons at 5 days after infection were fixed with 4% paraformaldehyde plus 4% sucrose in PBS for 20 min at room temperature. The cells were then incubated with 0.2% Triton X-100 in PBS for 10 min at room temperature, blocked with 10% normal donkey serum in PBS for 1 h at room temperature, and incubated with primary antibodies for 1 h at room temperature or at 4 °C overnight. After washing with PBS, the cells were incubated with conjugated secondary antibodies for 1 h at room temperature, washed with PBS, mounted using Mowiol mounting medium (w/v 24% glycerol, 9.6% Mowiol 4–88, 2.5% DABCO; v/v 36% ddH_2_O, 48% 0.2 M Tris pH 8.5), and imaged under a microscope.

### Time-lapse imaging

Time-lapse imaging was performed under the 60× objective lens of a live-cell observation system including a Nikon Ti-E-PFS microscope equipped with an Andor Zyla ultra-low noise CMOS camera and a Chamlide TC chamber. Images were acquired using MetaMorph Premier 7.8.6 software (Molecular Device), in the multidimensional acquisition mode. Hippocampal neurons on DIV10 were maintained in ACSF (110 mM NaCl, 5 mM KCl, 2 mM CaCl_2_, 0.8 mM MgCl_2_, 10 mM HEPES, 10 mM D-glucose, pH 7.4, and containing TTX and picrotoxin) and imaged every 3 s or 5 s for 5 min. COS7 cells at 1 day after transfection were maintained in CMEM and imaged every 2 s for 2 mins. For time-lapse imaging after nocodazole or cytochalasin D treatment, COS7 cells were imaged before treatment and then at 2-s intervals for 2 min once every 10 min.

### Surface biotinylation

Hippocampal neurons were washed thrice with B buffer (0.5 mM CaCl_2_ and 0.5 mM MgCl_2_) supplemented PBS and treated with 0.5 mg/mL sulfo-succinimidyl-6-(biotinamido) hexanoate (sulfo-NHS-LC-biotin; Pierce) in B buffer for 5 min at room temperature. The free sulfo-NHS-LC-biotin was removed by rapidly washing twice with 100 mM glycine in B buffer and then twice with B buffer. The biotinylated neurons were solubilized with 300 μL of radioimmunoprecipitation assay buffer (10 mM Tris, pH 7.4, 150 mM NaCl, 1 mM EDTA, 0.1% SDS, 1% Triton X-100, 1% sodium deoxycholate). After centrifugation at maximal speed for 15 min at 4 °C in a table-top centrifuge, a part of each supernatant was saved for estimation of total protein, and the remaining supernatants were incubated with 100 μL of a 50% slurry of NeutrAvidin beads (Pierce) for 1 h at 4 °C with constant rotation. After several washes, the biotinylated surface proteins were eluted from the NeutrAvidin beads in 100 μL of 1× SDS sample buffer and then analyzed using SDS-PAGE and western blotting.

### Hoechst staining

Hippocampal neurons were infected at DIV5 and at DIV 13 and then incubated for 6 h in either pH 7.4 or pH 6.0 HEPES buffer (10 mM HEPES, 10 mM glucose, 140 mM NaCl, 5 mM KCl, 2 mM CaCl_2_, 0.8 mM MgCl_2_). Chromatin condensation was detected through nuclear staining with Hoechst 33342 (Beyotime, C1026). Briefly, cultured neurons were washed once with PBS and fixed with 4% paraformaldehyde plus 4% sucrose in PBS for 15 min at room temperature. The neurons were then stained with Hoechst 33342 (5 μg/mL) for 15 min at room temperature, washed twice with PBS, and immediately mounted using Mowiol mounting medium, and imaged under a microscope.

### Image acquisition, processing, and data analysis

Fixed cells were examined under a Nikon Eclipse TE2000 inverted fluorescence microscope or an LSM510 microscope. Photographs were acquired using a monochrome low-noise cooled CCD camera controlled by MetaMorph software and edited using Adobe Photoshop. Cells examined under the LSM510 microscope were imaged using the built-in software. In a given experiment, identical acquisition parameters were used for each sample, and for quantification, at least 3 independent experimental repetitions were performed. Time-lapse images were analyzed by using ImageJ software. The PICK1 moving trajectories were produced by the Z-projection command in ImageJ. The PICK1 moving dynamic in neuron was analyzed firstly producing the kymograph after selecting and drawing line aligning with the axon. Single PICK1 particle walking distance was measured next from the kymograph image after selecting single PICK1 moving trace. The moving speed of PICK1 particle was then calculated by dividing the walking distance with the imaging duration. Protein axonal targeting and clustering were analyzed using MetaMorph software. Axons were distinguished from dendrites by morphological difference and MAP2 staining. The integrated intensity of protein signal in axons within a 200-μm diameter from the soma was divided by the intensity in dendrites to obtain the A/D ratio. Images were edited using Adobe Photoshop. Statistical comparisons were performed using SPSS independent *t* tests, one-way ANOVA Sidak test or nonparametric Kruskal-Wallis Test.

## Additional Information

**How to cite this article**: Xu, J. *et al.* Syntabulin regulates the trafficking of PICK1-containing vesicles in neurons. *Sci. Rep.* **6**, 20924; doi: 10.1038/srep20924 (2016).

## Supplementary Material

Supplementary Information

Supplementary movie 1

Supplementary movie 2

Supplementary movie 3

Supplementary movie 4

Supplementary movie 5

Supplementary movie 6

Supplementary movie 7

Supplementary movie 8

Supplementary movie 9

Supplementary movie 10
