# Supplementary material for: Syntabulin regulates the trafficking of PICK1-containing vesicles in neurons
Source: Sci Rep. 2016 Feb 12;6:20924. doi: 10.1038/srep20924 (PMC4751430; doi:10.1038/srep20924)

Syntabulin regulates the trafficking of PICK1-containing vesicles in neuron

Junyu Xu, Na Wang, Jian-hong Luo and Jun Xia

Supplementary figure legends:

Supplementary figure 1 Syntabulin C-terminal associates with mitotracker. COS7 cells expressing myc-tagged Syntabulin-C terminal fragment (Stb-Ct) were treated with DMSO or Nocodazole (5 μg/ml) for 30 mins, and then were stained with mitotracker and antibodies against myc and β-tubuin. Result showed that Stb-Ct colocalized with mitotracker, but not microtubule structures.

Supplementary figure 2 Axonal targeting of PICK1 and Syntabulin when expressed in hippocampal neurons. Hippocampal neurons were transfected with GFP-PICK1 alone (A) or together with myc-Syntabulin-FL (B) or myc-Syntabulin-Nt (C). Neurons were then stained with dendrite marker MAP2 and myc antibody. Arrowheads indicate axons, which are MAP2-signal free. Lower panels are the enlarged images around soma. Scale bars = 20 μm.

Supplementary figure 3 Generation and examination of Syntabulin antibody. (A) Strip test of syntabulin antiserum and purified antibody using rat brain homogenate. (B) Test of syntabulin antibody in myc-syntabulin transfected HEK293T cell lysates. (C) Mouse tissue expression of syntabulin detected by purified syntabulin antibody.

Supplementary movie 1 Time-lapse imaging of PICK1 and syntabulin in COS7 cells. COS7 cells were transfected with RFP-PICK1 (red) and GFP-syntabulin (green) and imaged using live-cell observation system for 2 mins with 2 sec interval.

Supplementary movie 2 Time-lapse imaging of PICK1 and syntabulin in COS7 cells before nocodazole treatment. COS7 cells were transfected with RFP-PICK1 (red) and GFP-syntabulin (green). Before nocodazole treatment, cells were imaged using live-cell observation system for 2 mins with 2 sec interval.

Supplementary movie 3 Time-lapse imaging of PICK1 and syntabulin in COS7 cells after nocodazole treatment. COS7 cells were transfected with RFP-PICK1 (red) and GFP-syntabulin (green). After nocodazole (5 μg/ml) treatment for 20 mins, the same cells in supplementary movie 2 were imaged using live-cell observation system for 2 mins with 2 sec interval.

Supplementary movie 4 Time-lapse imaging of PICK1 and syntabulin in COS7 cells before cytochalasin D treatment. COS7 cells were transfected with RFP-PICK1 (red) and GFP-syntabulin (green). Before cytochalasin D treatment, cells were imaged using live-cell observation system for 2 mins with 2 sec interval.

Supplementary movie 5 Time-lapse imaging of PICK1 and syntabulin in COS7 cells after cytochalasin D treatment. COS7 cells were transfected with RFP-PICK1 (red) and GFP-syntabulin (green). After cytochalasin D (2 μg/ml) treatment for 40 mins, the same cells in supplementary movie 2 were imaged using live-cell observation system for 2 mins with 2 sec interval.

Supplementary movie 6 Time-lapse imaging of PICK1 and syntabulin-SBD in COS7 cells. COS7 cells were transfected with RFP-PICK1 (red) and GFP-syntabulin-SBD (green) and imaged using live-cell observation system for 2 mins with 2 sec interval. No obvious movement of the vesicles were observed.

Supplementary movie 7 Time-lapse imaging of PICK1 in dendrites of hippocampal neurons. Neuronal cells were transfected with GFP-PICK1 on DIV5. The dendritic part of neuron were imaged using live-cell observation system for 5 mins with 5 sec interval on DIV10.

Supplementary movie 8 Time-lapse imaging of PICK1 in axon of hippocampal neurons. Neuronal cells were transfected with GFP-PICK1 on DIV5. The axons of neuron were imaged using live-cell observation system for 5 mins with 3 sec interval on DIV10.

Supplementary movie 9 Time-lapse imaging of PICK1 in axon of hippocampal neurons when co-expressed with syntabulin-FL. Neuronal cells were transfected with GFP-PICK1 and myc-syntabulin-FL on DIV5. The axons of neuron were imaged using live-cell observation system for 5 mins with 3 sec interval on DIV10.

Supplementary movie 10 Time-lapse imaging of PICK1 in axon of hippocampal neurons when co-expressed with syntabulin-Nt. Neuronal cells were transfected with GFP-PICK1 and myc-syntabulin-Nt on DIV5. The axons of neuron were imaged using live-cell observation system for 5 mins with 3 sec interval on DIV10.

Supplementary figures:

Supplementary figure 1

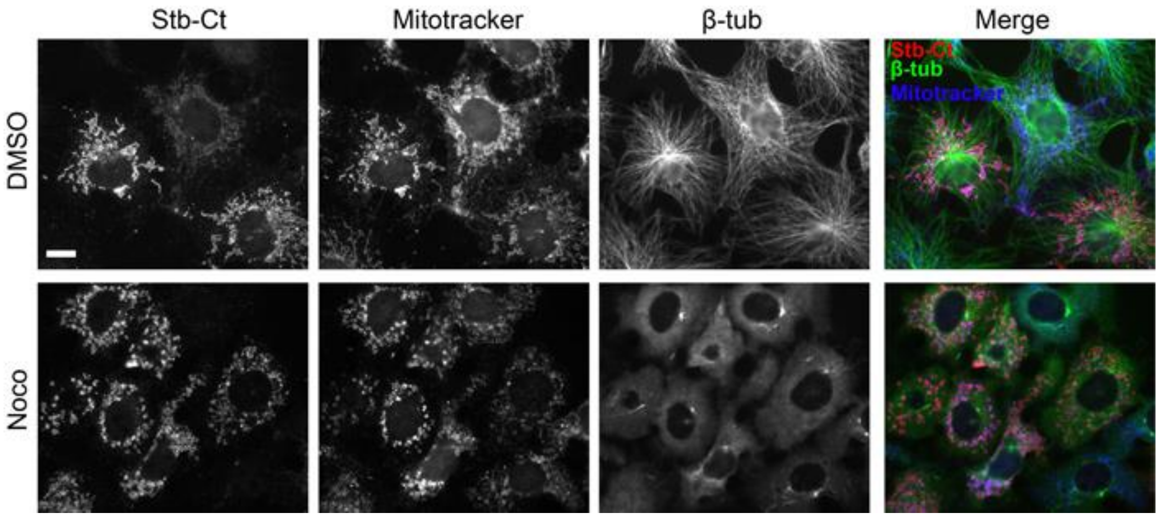

Supplementary figure 2

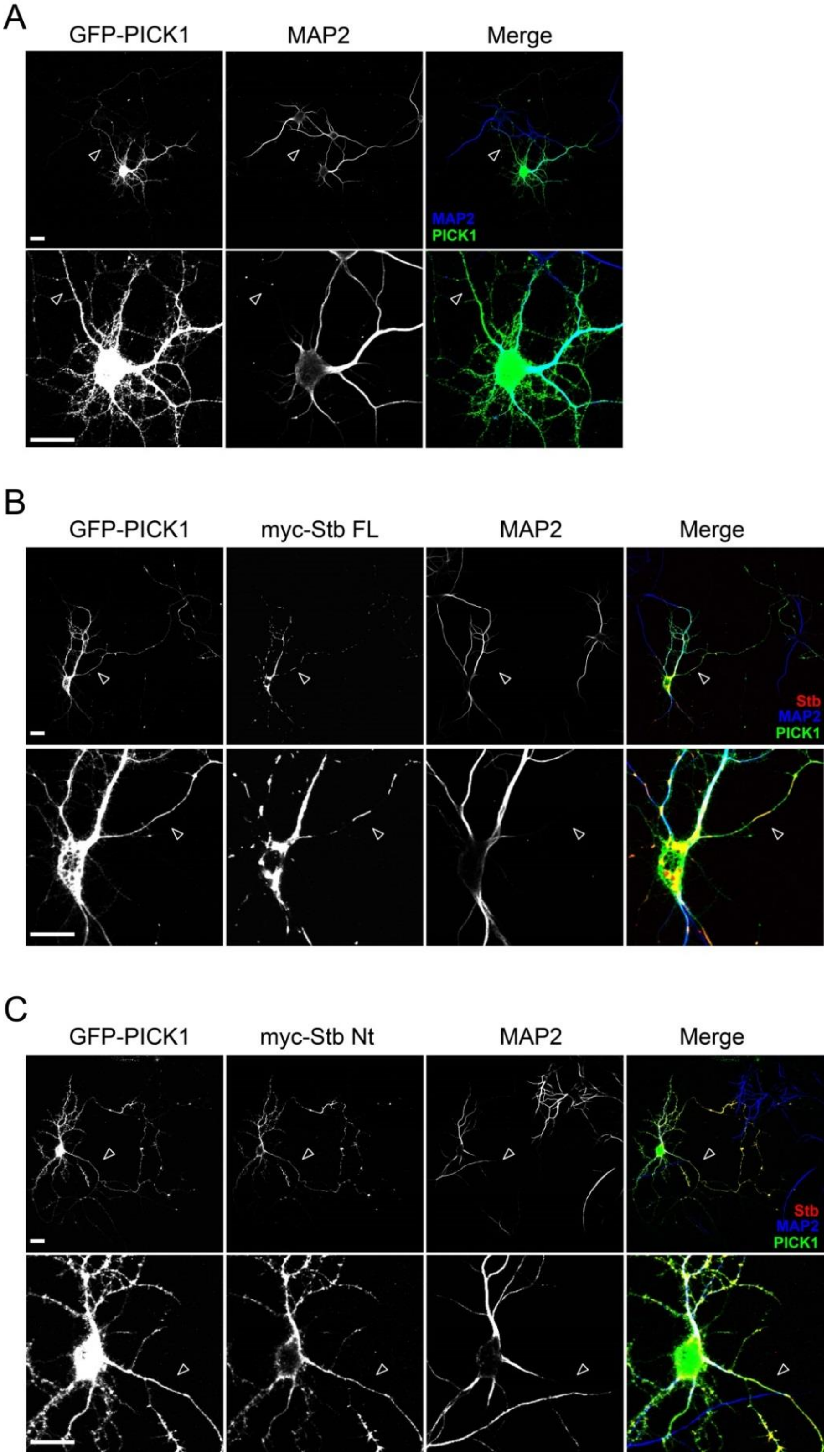

Supplementary figure 3

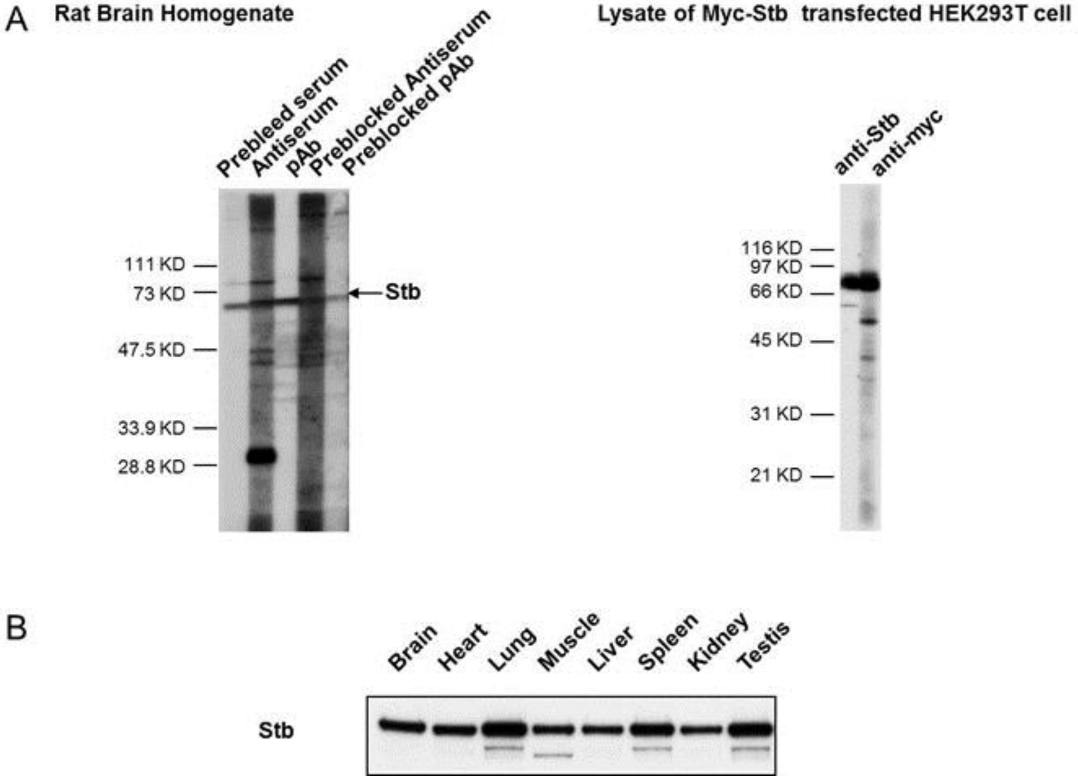

Supplement: Supplementary Information [file srep20924-s1.pdf]
